# *Leptospira* infection and shedding in dogs in Thailand

**DOI:** 10.1186/s12917-020-2230-0

**Published:** 2020-03-17

**Authors:** Kerstin Altheimer, Prapaporn Jongwattanapisan, Supol Luengyosluechakul, Rosama Pusoonthornthum, Nuvee Prapasarakul, Alongkorn Kurilung, Els M. Broens, Jaap A. Wagenaar, Marga G. A. Goris, Ahmed A. Ahmed, Nikola Pantchev, Sven Reese, Katrin Hartmann

**Affiliations:** 1 Clinic of Small Animal Medicine, Centre for Clinical Veterinary Medicine, LMU Munich, Veterinaerstrasse 13, 80539 Munich, Germany; 2 Department of Veterinary Medicine, Faculty of Veterinary Science, Chulalongkorn University, Bangkok, Thailand; 3 Department of Veterinary Microbiology, Faculty of Veterinary Science, Chulalongkorn University, Bangkok, Thailand; 4 Department of Infectious Diseases and Immunology, Faculty of Veterinary Medicine, Utrecht University, Utrecht, the Netherlands; 5 Wageningen Bioveterinary Research, Lelystad, the Netherlands; 6 OIE and National Collaborating Centre for Reference and Research on Leptospirosis, Department of Medical Microbiology, Academic Medical Center, Amsterdam, the Netherlands; 7 IDEXX Laboratories, Ludwigsburg, Germany; 8 Department of Veterinary Sciences, Institute of Anatomy, Histology and Embryology, LMU Munich, Munich, Germany

**Keywords:** Canine, Culture, Dogs, ELISA, *Leptospira*, MAT, PCR, Risk factors, Seroprevalence, Zoonosis

## Abstract

**Background:**

Leptospirosis is a widespread zoonosis and has been recognized as a re-emerging infectious disease in humans and dogs, but prevalence of *Leptospira* shedding in dogs in Thailand is unknown. The aim of this study was to determine urinary shedding of *Leptospira* in dogs in Thailand, to evaluate antibody prevalence by microscopic agglutination test (MAT) and enzyme-linked immunosorbent assay (ELISA), and to assess risk factors for *Leptospira* infection.

In Northern, Northeastern, and Central Thailand, 273 stray (*n* = 119) or client-owned (*n* = 154) dogs from rural (*n* = 139) or urban (*n* = 134) areas were randomly included. Dogs that had received antibiotics within 4 weeks prior to sampling were excluded. No dog had received vaccination against *Leptospira*. Urine was evaluated by real-time polymerase chain reaction (PCR) specific for *lipL32* gene of pathogenic *Leptospira*. Additionally, urine was cultured for 6 months in Ellinghausen-McCullough-Johnson-Harris (EMJH) medium. Antibodies were measured by ELISA and MAT against 24 serovars belonging to 15 serogroups and 1 undesignated serogroup. Risk factor analysis was performed with backwards stepwise selection based on Wald.

**Results:**

Twelve of 273 (4.4%; 95% confidence interval (CI): 2.0–6.8%) urine samples were PCR-positive. In 1/273 dogs (0.4%; 95% CI: 0.01–1.1%) *Leptospira* could be cultured from urine. MAT detected antibodies in 33/273 dogs (12.1%; 95% CI: 8.2–16.0%) against 19 different serovars (Anhoa, Australis, Ballum, Bataviae, Bratislava, Broomi, Canicola, Copenhageni, Coxi, Grippotyphosa, Haemolytica, Icterohaemorrhagiae, Khorat, Paidjan, Patoc, Pyrogenes, Rachmati, Saxkoebing, Sejroe). In 111/252 dogs (44.0%; 95% CI: 37.9–50.2%) immunoglobulin M (IgM) and/or immunoglobulin G (IgG) antibodies were found by ELISA. Female dogs had a significantly higher risk for *Leptospira* infection (*p* = 0.023).

**Conclusions:**

*Leptospira* shedding occurs in randomly sampled dogs in Thailand, with infection rates comparable to those of Europe and the USA. Therefore, the potential zoonotic risk should not be underestimated and use of *Leptospira* vaccines are recommended.

## Background

Leptospirosis is categorized as a neglected zoonotic disease, affecting both humans and animals [1]. The disease is caused by spiral-shaped, gram-negative spirochetes of the genus *Leptospira*. To date, there are more than 260 different *Leptospira* serovars worldwide. Almost all mammalian species and marsupials can become renal carriers, and human infections originate from animal carriers [2]. The importance of the infection for public health and veterinary medicine is significant, and the impact of animal leptospirosis probably exceeds that in human [3]. In Thailand, human leptospirosis is classified as an emerging infectious disease with an outbreak peak of 14,285 cases in the year 2000 [4]. Recent data from Thailand even demonstrate a nationwide increase in 2017 compared to 2015–2016. In total, 3156 leptospirosis cases and 57 fatalities were registered in 2017, with a morbidity rate of 4.8 and a mortality rate of 0.09 per 100,000 population. Most cases were reported from Northeastern Thailand [5]. Moreover, an alarmingly high antibody prevalence of 89.1% (205/230) was documented in stray dogs from Bangkok [6] (Table 1), and the constantly increasing number of stray dogs has become a public health issue in Thailand [11]. Dogs, especially strays, are considered an important reservoir of *Leptospira*, and thus play a major role in human infections [12–16]. In addition, “dog ownership” was identified as a potential risk factor for humans [17–22]. Worldwide studies showed a prevalence of urinary shedding of *Leptospira* in dogs between 0.2 and 31.1% by PCR [23–37]. Shedding can also occur in healthy dogs [23, 25, 31–33, 35, 37]. Thus, dogs recently gained interest as potential source of human infection.

**Table 1:** Prevalence of microscopic agglutination test (MAT) antibodies of dogs tested at various regions in Thailand

Region of Thailand	Number of dogs sampled	MAT cut-off	Antibody prevalence	Most common seroreactivity	Study reference
Chaiyaphum province, Northeastern Thailand	47	≥1:100	4.3% (2/47)	Autumnalis	[7]
Mahasarakham province, Northeastern Thailand	55	≥1:100	10.9% (6/55)	Canicola	[8]
Chiang Mai, Northern Thailand	210	≥1:20	11.0% (23/210)	Bataviae, Canicola, Bratislava, Icterohaemorrhagiae, Ballum, Djasiman, Javanica, Mini, Sejroe	[9]
Nakhon Pathom province, Central Thailand	153	≥1:50	57.5% (88/153)	Tarassovi, Ranarum, Saigon, Bratislava, Copenhageni, Patoc, Bangkok, Sejroe, Autumnalis, Sarmin, Canicola	[10]
Bangkok, Central Thailand	230	≥1:100	89.1% (205/230)	Bataviae, Patoc, Tarassovi, Sejroe, Shermani, Autumnalis, Ranarum, Sarmin, Grippotyphosa, Hebdomadis, Manhao, Pomona, Louisiana, Bratislava, Cynopteri	[6]

There are no comprehensive studies on *Leptospira* urinary shedding in dogs in Thailand, although several studies demonstrated presence of antibodies against *Leptospira* in 4.3 to 89.1% of dogs [6–10] (Table 1). Moreover, a recently published small study from Thailand detected *Leptospira* in the urine of 10.3% (6/58) asymptomatic dogs by *rrs* nested PCR [32]. Therefore, the aims of the present study were to determine *Leptospira* urinary shedding prevalence by real-time polymerase chain reaction (PCR), to culture *Leptospira* from urine, to evaluate *Leptospira* antibody prevalence by microscopic agglutination test (MAT) and by enzyme-linked immunosorbent assay (ELISA) differentiating immunoglobulin M (IgM) and immunoglobulin G (IgG) antibodies, and to assess risk factors associated with *Leptospira* infection in dogs in Thailand.

## Results

### Prevalence of *Leptospira* urinary shedding

In 12/273 dogs, DNA from pathogenic *Leptospira* was amplified from urine; thus, prevalence of urinary *Leptospira* DNA shedding was 4.4% (95% CI: 2.0–6.8%). Five of 12 PCR-positive dogs (41.7%) were client-owned and 7/12 (58.3%) were stray. Eight shedders were of rural origin (66.7%); 4/12 (33.3%) came from urban areas (Table 2). MAT was positive in 4/12 (33.3%) PCR-positive dogs; 9/12 (75.0%) PCR-positive dogs had detectable antibodies in IgM/IgG ELISA.

**Table 2:** Characteristics of the 12 dogs shedding *Leptospira* determined by real-time PCR in urine

Signalment	Status	Origin	Reason for presentation	Physical examination	Urine PCR Ct value	Urine culture	MAT (cut-off: ≥1:20)	IgM/IgG ELISA (cut-off: ≥1:320)
4 y, mix, f, i	client-owned	Amnat Charoen, rural	neutering	unremarkable	28.3	neg	1:640 Sejroe 1:320 Saxkoebing 1:20 Haemolytica	IgM 1:1280 IgG 1:640
1 y, mix, f, i	client-owned	Amnat Charoen, rural	neutering	unremarkable	38.0	neg	neg	neg
2 y, mix, f, i	client-owned	Lamphun, rural	neutering	enlarged Lnn. mandibulares	36.8	neg	1:80 Sejroe 1:40 Saxkoebing	IgM neg IgG 1:640
~ 3 y, mix, m, i	stray	Pathum Thani, rural	neutering	unremarkable	33.1	neg	neg	neg
~ 2 y, mix, f, i	stray	Pathum Thani, rural	neutering	mildly increased inspiratory lung sounds	34.5	neg	neg	IgM 1:320 IgG neg
~ 6 m, mix, f, i	stray	Pathum Thani, rural	neutering	unremarkable	29.0	pos	neg	IgM 1:320 IgG neg
~ 2 y, mix, f, i	stray	Samut Songkhram, rural	neutering	unremarkable	32.9	neg	neg	IgM 1:320 IgG neg
~ 3 y, mix, f, i	stray	Samut Songkhram, rural	neutering	mildly increased inspiratory lung sounds, enlarged Lnn. mandibulares	31.4	neg	neg	IgM 1:640 IgG neg
1 y, mix, f, i	client-owned	Nakohn Ratchasima, urban	neutering	unremarkable	30.0	neg	neg	IgM 1:320 IgG neg
3 y, poodle, m, i	client-owned	Nakhon Ratchasima, urban	neutering	enlarged Lnn. mandibulares	36.7	neg	1:40 Icterohaemorrhagiae	IgM neg IgG 1:2560
~ 3 y, mix, m, i	stray	Bangkok, urban	neutering	unremarkable	28.3	neg	1:640 Bataviae 1:80 Paidjan	IgM 1:2560 IgG 1:640
~ 4 y, mix, f, i	stray	Bangkok, urban	neutering	unremarkable	>40.0	neg	neg	neg

*y* years, *m* months, *mix* mixed breed, *f* female, *m* male, *i* intact, *Lnn*. lymph nodes, *PCR* polymerase chain reaction, *Ct value* threshold cycle, *neg* negative, *pos* positive, *MAT* microscopic agglutination test, *ELISA* enzyme-linked immunosorbent assay, *IgM* immunoglobulin M, *IgG* immunoglobulin G

**Fig. 1:**
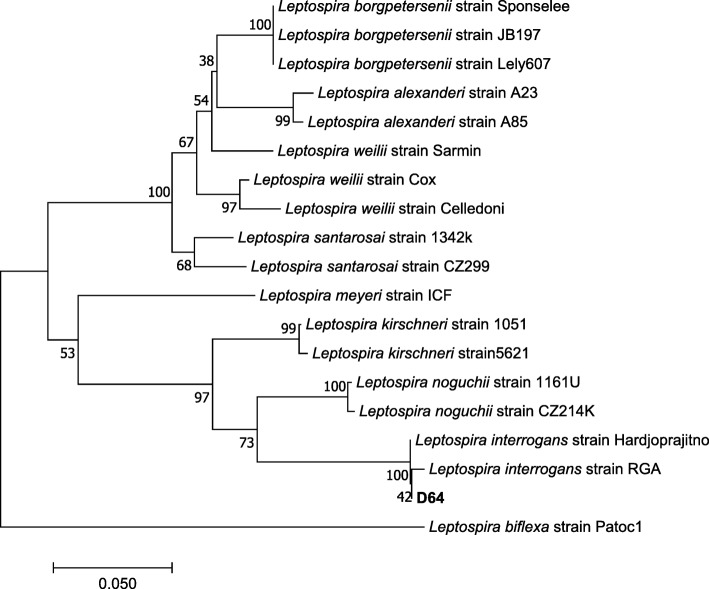
Evolutionary relationships of taxa. The evolutionary history was inferred using the Neighbor-Joining method. The optimal tree with the sum of branch length = 0.76583659 is shown. The percentage of replicate trees in which the associated taxa clustered together in the bootstrap test (1000 replicates) are shown next to the branches. The tree is drawn to scale, with branch lengths in the same units as those of the evolutionary distances used to infer the phylogenetic tree. The evolutionary distances were computed using the Maximum Composite Likelihood method and are in the units of the number of base substitutions per site. The analysis involved 19 nucleotide sequences. Codon positions included were 1st + 2nd + 3rd + noncoding. All positions containing gaps and missing data were eliminated. There were a total of 245 positions in the final dataset. Evolutionary analyses were conducted in MEGA 7. The bar indicates 0.050 estimated substitution per sequence position. Dog D64 of the present study clusters within the genomospecies *Leptospira interrogans*

Urine of all 273 dogs was cultured for 6 months. In only 1 urine culture (0.4%; 95% CI: 0.01–1.1%), *Leptospira* were growing after an incubation period of 3 months. All other 272 cultures remained negative after 6 months. The dog with the positive culture was also positive in urine PCR (Table 2). In ELISA, this dog had IgM antibodies of 1:320, but no IgG antibodies. No antibodies were found by MAT. Phylogenetic analysis based on *secY* sequencing showed that this *Leptospira* strain belonged to the pathogenic genospecies *Leptospira interrogans* (Fig. 1).

### Antibody prevalence

Anti-leptospiral antibodies were detected in MAT in 33/273 dogs (12.1%; 95% CI: 8.2–16.0%). Antibodies to more than 1 serovar were found in 15/33 MAT-positive dogs (45.5%). Antibodies were detected against 19 serovars belonging to 12 serogroups. The most common serogroup was Sejroe (4.4%), followed by Icterohaemorrhagiae (3.7%), Bataviae (2.9%), and Canicola (2.6%). MAT titers ranged from 1:20 to 1:640 (Table 3). A very high MAT titer of 1:640 was only found in 2 dogs against serogroup Bataviae (serovar Bataviae) and Sejroe (serovar Sejroe). These dogs also had high IgM and IgG antibodies in ELISA. The dog with high antibodies against serogroup Sejroe had IgM titer of 1:1280 and IgG titer of 1:640. The dog with MAT titer of 1:640 against serogroup Bataviae had IgM titer of 1:2560 and IgG titer of 1:640. Both dogs were urine PCR-positive (Table 2) but not positive in urine culture.

**Table 3:** Number and percentage of microscopic agglutination test- (MAT-) positive results among 273 dogs^a^

Serogroup	Serovar	Number of dogs with respective MAT titers						Total number of dogs with MAT titers ≥1:20	Percentage of dogs with MAT titers ≥1:20 (95% CI)
		1:20	1:40	1:80	1:160	1:320	1:640		
Australis	Australis	2	0	0	0	0	0	2	0.7 (0.0–1.7)
	Bratislava	2	0	0	1	0	0	3	1.1 (0.0–2.3)
Autumnalis	Autumnalis	0	0	0	0	0	0	0	0.0
	Rachmati	2	0	0	0	0	0	2	0.7 (0.0–1.7)
Ballum	Ballum	3	0	1	0	0	0	4	1.5 (0.0–2.9)
Bataviae	Bataviae	0	4	0	0	0	1	5	1.8 (0.2–3.4)
	Paidjan	1	1	1	0	0	0	3	1.1 (0.0–2.3)
Canicola	Broomi	4	0	0	0	0	0	4	1.5 (0.0–2.9)
	Canicola	0	2	1	0	0	0	3	1.1 (0.0–2.3)
Celledoni	Anhoa	1	0	0	0	0	0	1	0.4 (0.0–1.1)
	Celledoni	0	0	0	0	0	0	0	0.0
Cynopteri	Cynopteri	0	0	0	0	0	0	0	0.0
Djasiman	Djasiman	0	0	0	0	0	0	0	0.0
Grippotyphosa	Grippotyphosa	3	0	0	0	0	0	3	1.1 (0.0–2.3)
Icterohaemorrhagiae	Copenhageni	1	1	1	1	0	0	4	1.5 (0.0–2.9)
	Icterohaemorrhagiae	0	3	2	1	0	0	6	2.2 (0.5–3.9)
Javanica	Coxi	0	0	1	0	0	0	1	0.4 (0.0–1.1)
Pomona	Pomona	0	0	0	0	0	0	0	0.0
Pyrogenes	Pyrogenes	3	2	0	0	0	0	5	1.8 (0.2–3.4)
Sejroe	Haemolytica	1	1	0	0	0	0	2	0.7 (0.0–1.7)
	Saxkoebing	2	1	0	3	1	0	7	2.6 (0.7–4.4)
	Sejroe	0	1	1	0	0	1	3	1.1 (0.0–2.3)
Semaranga	Patoc	1	1	0	0	0	0	2	0.7 (0.0–1.7)
Undesignated	Khorat	1	1	0	0	0	0	2	0.7 (0.0–1.7)
Total		27	18	8	6	1	2	62	22.7 (17.7–27.7)

^**a**^Antibodies against more than one serovar were detected in 15/33 MAT-positive dogs

*CI* confidence interval

IgM and IgG ELISA was performed in 252/273 dogs. In 17/273 dogs, only IgM ELISA was performed and in 4/273 dogs, neither IgM nor IgG ELISA was performed due to limited amount of serum. In 111/252 dogs (44.0%; 95% CI: 37.9–50.2%), either IgM and/or IgG antibodies were detectable. Comparing results of ELISA to MAT, 100/252 dogs (39.7%; 95% CI: 33.6–45.7%) showed discrepant results. Of 141 ELISA-negative dogs, 9 dogs were positive in MAT. Presence of both IgM and IgG antibodies (≥1:320) was found in 41 dogs of which only 14/41 were also MAT-positive (≥1:20), resulting in a discrepancy of 65.9%. Of 111 ELISA-positive dogs, 86 dogs (77.5%) were completely negative in all other diagnostic assays (PCR, urine culture, MAT).

### Risk factor analysis

Risk factors associated with *Leptospira* infection in dogs are illustrated in Table 4. In univariate analysis, female dogs (odds ratio [OR] 1.910; 95% confidence interval [95% CI] 1.138–3.204; *p* = 0.014) and dogs with no cattle contact (OR 4.697; 95% CI 1.382–15.969; *p* = 0.013) were significantly more commonly infected with *Leptospira* than male dogs and dogs with cattle contact. Only the category “female sex” (OR 1.890; 95% CI 1.092–3.270; *p* = 0.023) proved to be significantly associated with *Leptospira*-infected dogs after backwards stepwise selection based on Wald. None of the investigated parameters was significantly associated with presence of antibodies against *Leptospira* determined by MAT (see Additional file 1: Table S1), or with urinary shedding of *Leptospira* detected by PCR (see Additional file 2: Table S2).

**Table 4:** Risk factor analysis for dogs being *Leptospira*-infected (PCR- and/or ELISA- and/or MAT-positive)

Variable	Total dogs	Categories	Number of dogs tested	*Leptospira-*positive	*Leptospira*-negative	Univariate analysis			Multivariate analysis (*n* = 242)		
				(%)	(%)	Odds ratio	95% CI	*p*	Odds ratio	95% CI	*p*
Age	242	<1 year	36	18 (50.0)	18 (50.0)	1.000	0.428–2.339	1.000	^a^	^a^	^a^
		1.0–1.9 years	64	35 (54.7)	29 (45.3)	1.207	0.580–2.513	0.709			
		2.0–2.9 years	45	22 (48.9)	23 (51.1)	0.957	0.431–2.125	1.000			
		3.0–3.9 years	52	26 (50.0)	26 (50.0)	Reference					
		4.0–5.9 years	28	13 (46.4)	15 (53.6)	0.867	0.345–2.176	0.817			
		≥6 years	17	8 (47.1)	9 (52.9)	0.889	0.297–2.661	1.000			
Breed	273	mix	266	132 (49.6)	134 (50.4)	1.313	0.288–5.982	0.725	^a^	^a^	^a^
		pure breed	7	3 (42.9)	4 (57.1)						
Sex	273	female	185	101 (54.6)	84 (45.4)	1.910	1.138–3.204	**0.014**	1.890	1.092–3.270	**0.023**
		male	88	34 (38.6)	54 (61.4)						
Neutering status	273	intact	270	134 (49.6)	136 (50.4)	1.971	0.177–21.992	1.000	^a^	^a^	^a^
		neutered	3	1 (33.3)	2 (66.7)						
Weight	175	5–11 kg	48	23 (47.9)	25 (52.1)	0.595	0.291–1.218	0.202			
		12–17 kg	84	51 (60.7)	33 (39.3)	Reference					
		≥18 kg	43	28 (65.1)	15 (34.9)	1.208	0.562–2.595	0.701			
Origin	273	client-owned	154	74 (48.1)	80 (51.9)						
		stray	119	61 (51.2)	58 (48.8)	1.137	0.705–1.835	0.627	^a^	^a^	^a^
Environment	273	urban	134	73 (54.5)	61 (45.5)	1.486	0.923–2.395	0.103	^a^	^a^	^a^
		rural	139	62 (45.6)	77 (54.4)						
Free-running/roaming allowed	180	yes	174	90 (51.7)	84 (48.3)						
		no	6	5 (83.3)	1 (16.7)	4.667	0.534–40.773	0.164			
Staying outdoors >50%	168	yes	148	79 (53.3)	69 (46.7)						
		no	20	11 (55.0)	9 (45.0)	1.068	0.418–2.728	1.000			
Bathing in water	32	yes	13	8 (61.5)	5 (38.5)						
		no	19	12 (63.3)	7 (36.7)	1.0714	0.250–4.591	1.000			
Drinking out of puddles	34	yes	13	8 (61.5)	5 (38.5)						
		no	21	13 (61.9)	8 (38.1)	1.016	0.245–4.213	1.000			
Contact with rodents	33	yes	22	14 (63.6)	8 (36.4)	1.000	0.222–4.502	1.000			
		no	11	7 (63.6)	4 (36.4)						
Eating rodents	33	yes	6	6 (100.0)	0 (0.0)	10.484	0.537–204.643	0.065			
		no	27	15 (55.5)	12 (44.5)						
Consumption of raw meat	40	yes	12	7 (58.3)	5 (41.7)						
		no	28	17 (60.7)	11 (39.3)	1.104	0.279–4.369	1.000			
Hunting dog	273	yes	0	0 (0.0)	0 (0.0)						
		no	273	135 (49.5)	138 (50.5)	1.0221	0.020–51.886	1.000			
Contact with cats	50	yes	24	15 (62.5)	9 (37.5)	1.667	0.539–5.153	0.375			
		no	26	13 (50.0)	13 (50.0)						
Contact with other dogs	176	yes	175	90 (51.4)	85 (48.6)						
		no	1	1 (100.0)	0 (0.0)	2.834	0.114–70.532	1.000			
Contact with cattle	58	yes	16	6 (37.5)	10 (62.5)						
		no	42	31 (73.8)	11 (26.2)	4.697	1.382–15.969	**0.013**			
Contact with pigs	58	yes	1	1 (100.0)	0 (0.0)	1.767	0.069–45.335	1.000			
		no	57	36 (63.2)	21 (36.8)						

Univariate and multivariate analysis for risk factors associated with positivity in at least one diagnostic *Leptospira* test (*n* = 135/273): urine PCR, MAT (cut-off: ≥1:20), IgM ELISA, and IgG ELISA (cut-off: ≥1:320). For multivariate analysis, backward stepwise selection based on Wald was performed for the following parameters: age, breed, sex, neutering status, origin, and environment

^a^Variable was eliminated in backward stepwise selection

Significant *p*-values are shown in bold

*PCR* polymerase chain reaction, *ELISA* enzyme-linked immunosorbent assay, *MAT* microscopic agglutination test, *CI* confidence interval, *p p*-value

## Discussion

This is the first comprehensive study investigating urinary shedding of *Leptospira* in dogs in Thailand, revealing a shedding prevalence of 4.4% in dogs in Northern, Northeastern, and Central Thailand. The results of this study are of high importance, because *Leptospira* shedding is a potential infection risk for people in contact with infected dogs. Moreover, shedding dogs contribute to *Leptospira* spread in the environment [15]. Shedding of *Leptospira* in dogs starts at day 7–10 after infection and lasts for 4 to 6 weeks [38–40], sometimes even over several years [38]. Time period and quantity differ individually and vary between infecting serogroups [15, 41]. The shedding prevalence in the present study of 4.4% appears rather low, but the true shedding prevalence might be underestimated because only 12/273 dogs were shedding, while anti-leptospiral antibodies were found in 33/273 (12.1%) dogs in MAT, and in 111/252 (44.0%) dogs in ELISA. Thus, almost half of the dogs had been infected at least once in their life, as none of them had ever received a *Leptospira* vaccine.

The shedding prevalence is comparable to other investigations. In Ireland, 7.1% (37/525) of dogs were shedding [29], 8.2% (41/500) in the USA [31], 31.1% in Iran [37], and 19.8% of the dogs in Brazil [35]. In a German study, 1.5% (3/200) of healthy dogs were shedding *Leptospira* [25], and in Switzerland, the shedding prevalence was 0.2% (1/408) [23]. These low European prevalences are probably due to a broader vaccine-induced immunity in the dog population. Considering the fact that human leptospirosis is endemic in Thailand with its hot humid climate, a much higher shedding prevalence would have been expected in the present survey. Possible explanations are that leptospirosis is more a seasonal disease and no natural disaster, e.g. flooding, which is well documented to enable leptospirosis outbreaks particularly during the rainy season in Thailand, occurred at the time of sampling for the present study [42, 43]. A recently published study from Thailand detected a higher shedding prevalence of 10.3% in dogs, as *Leptospira* were detected in the urine of 6/58 asymptomatic dogs. All these dogs came from Nan province, a rural area in Northern Thailand where leptospirosis is known to be endemic. These urine PCR-positive dogs lived in close contact with livestock and were also used for hunting armadillo and bamboo rats [32]. These facts could explain the higher shedding prevalence found in Nan province compared to the shedding prevalence in the present study.

Culturing *Leptospira* is not a very sensitive method. The low pH of dog urine kills *Leptospira* rapidly [15]. Thus, a fast transfer into culture medium is mandatory and was performed in the present study. Nevertheless, only 1/273 (D64) culture samples was positive. Phylogenetic *secY* analysis revealed that this dog was infected with pathogenic *Leptospira interrogans*. This dog was also urine PCR-positive with a PCR *threshold cycle* (Ct) value of 29.0, and had IgM antibodies of 1:320 without IgG antibodies in ELISA or MAT antibodies implying that the infection had been acquired very recently. As culturing *Leptospira* from urine is not a sensitive method, it is not surprising that no other PCR-positive dog was culture-positive. Another important aspect which could explain the failure to grow *Leptospira* might be related to a relatively high Ct value of ≥30 in 9/12 PCR-positive dogs, indicating a rather low quantity of excreted *Leptospira* DNA.

Two different antibody tests were performed. MAT is regarded as the gold standard, but its sensitivity is low. MAT is only at best serogroup-specific and cannot exactly discriminate on serovar level [15]. As no dog in this study had been vaccinated against *Leptospira*, vaccine-induced interference can be excluded, and the anti-leptospiral antibodies found in 33 dogs (12.1%) were related to exposure. A similar MAT antibody reactivity was found in an older survey in Thailand, revealing an antibody prevalence of 11.0% [9], and a study recently conducted in Northeastern Thailand showed similar results with 10.9% [8]. However, a study on stray dogs in Bangkok in 2009 detected a much higher MAT antibody prevalence of 89.1% (Table 1). All stray dogs of that study were sampled in the center of Bangkok in Buddhist monasteries with close contact to rats which are reservoir hosts of several *Leptospira* species [6]. There might be a difference in exposure rates of stray dogs and client-owned dogs of which a high number was included in the present study. Client-owned dogs are normally fed by their owners, whereas stray dogs are more likely to hunt rats and mice and thus, to become *Leptospira*-infected.

In the present study, almost half of the MAT-positive dogs (15/33) had antibodies to at least more than one serovar (Table 3) which is presumably due to cross-reactivity which can occur on serovar or even serogroup level [44]. Cross-reactions with other infections, the onset of an acute infection accompanied by a rise in antibodies, or persisting antibodies in a chronic course of infection might be reasons for low antibody titers in MAT [3]. In the present study, the most common reactivity was against serogroup Sejroe, which is present in *Rattus rattus*, *Bandicota indica*, and *Bandicota savilei* in Thailand [45]. This highlights the importance of transmission from rodents. The second frequently reactive serogroup in the present study was Icterohaemorrhagiae which is most commonly involved in human infections worldwide [46], indicating that dogs play an important role in human infection, but rats are also known reservoirs [15]. A high MAT titer could be detected in only 2/273 dogs with a titer of 1:640 against Serogroup Sejroe and Bataviae, respectively, that were also positive in urine PCR and had high IgM and IgG titers in ELISA. These results are consistent with an acute but subclinical infection in both dogs, as their physical examination was unremarkable (Table 2).

Only 12.1% dogs had antibodies in MAT in the present study, but it is possible that serovars might have been missed due to an incomplete MAT panel. MAT is also commonly still negative in early infections, while IgM ELISA already reveals positive results [47–51]. This is in line with the finding that only 2/50 dogs that were IgM ELISA-positive and IgG ELISA-negative also had detectable MAT antibodies. MAT and ELISA showed a poor agreement indicating a higher sensitivity of ELISA in early infection [47, 49–52]. Another reason might be a lower specificity of ELISA compared to MAT. In humans living in endemic countries, persistence of IgM antibodies for many months or even years after infection and repeated exposure to nonpathogenic *Leptospira* was suggested as an explanation for positive IgM results in healthy humans [53–56].

When comparing antibody findings to PCR results, 9/12 shedders were also ELISA-positive, whereas only 4/12 shedders had antibodies in MAT. The discrepancy between urine PCR and antibody detection is in accordance with a study on 500 dogs in which 41 dogs were shedding *Leptospira*, while MAT was only positive in 9 dogs [31]. Shedding can occur before MAT antibodies are present [31, 57–59]. Another explanation could be immunosuppression or ongoing shedding in chronically infected dogs in which the level of IgM and IgG antibodies had already decreased below detection threshold [15, 60].

In the present study, female sex proved to be significantly associated with *Leptospira* infection in multivariate analysis (Table 4). This finding is in contrast to results of other surveys in which male dogs were at higher risk [61–65] and is also in contrast to a published meta-analysis in which the variable “male dogs” was a significant factor [66]. Interestingly, no further parameters in the present study were significantly associated with *Leptospira* infection in multivariate analysis. Other studies found a significant predisposition of urban dogs compared to rural living dogs attributed to a higher exposure to wildlife reservoir hosts [66–70]. One could also expect a significantly higher risk of infection for stray dogs. However, client-owned outdoor and stray dogs reside in very similar living environments in both (sub)urban and rural settings in Thailand, and contact to wild-living reservoir hosts occurs in urban and rural areas of Thailand. Sanitation and hygienic standards, including rodent control, might be comparable in both environments. Thus, both stray dogs and client-owned dogs might equally contribute to environmental contamination and potential transmission of *Leptospira.* Moreover, access to *Leptospira*-contaminated water sources exists in both environments.

## Conclusions

In conclusion, shedding prevalence of *Leptospira* in dogs taken at random in Thailand was low and not as high as expected for a tropical country. Still, in order to reduce the risk of infection and shedding, vaccines against *Leptospira* for dogs that are available in Thailand should be recommended as core vaccination, at least for client-owned outdoor dogs. Molecular genetic assays would be of particular importance in order to determine *Leptospira* strains in dogs in Thailand and globally.

## Methods

### Dogs

In total, 273 randomly selected dogs from rural (*n* = 139) and urban (*n* = 134) areas with outdoor access from Northern, Northeastern, and Central Thailand were included. Dogs were presented for either spaying/neutering or for rabies vaccination at public and private castration and vaccination programs. Dogs living indoors only and dogs treated with antimicrobials within the last 4 weeks prior to sampling were excluded. The study population consisted of 266 mixed-breed and 7 pure breed dogs; 154 dogs were client-owned and 119 stray; 185 dogs were female (2 spayed) and 88 male (1 neutered). No dog had received vaccination against *Leptospira*. After spaying/neutering and/or rabies vaccination, all client-owned dogs returned to their owners. Of the stray dogs, 77 (64.7%) were brought back and released to the territories where they had been trapped by private and public services for spaying/neutering and rabies vaccination. Forty-two of 119 stray dogs (35.3%) were admitted to governmental dog shelters (no-kill shelters) after spaying/neutering and rabies vaccination. None of the dogs were euthanized.

### Sample collection

Blood samples were obtained via puncture of the cephalic or femoral vein. Serum samples were stored at −20 °C until further processing. Urine samples were collected by ultrasound-guided cystocentesis (sample volumes of 1.5 ml to 16.0 ml) and stored at 4 °C for a maximum of 24 h, and then transferred into 1.5 ml Eppendorf tubes (Eppendorf AG, Hamburg, Germany). Tubes were centrifuged (14,000 x g, room temperature) for 15 min; supernatants were discarded. Pellets were washed with phosphate buffered saline (PBS) and transferred into Eppendorf tubes. A second centrifugation (14,000 x g, room temperature) was performed for 15 min, supernatants were discarded, and pellets were resuspended in 180 μl animal tissue lysis (ATL) buffer (Qiagen, Hilden, Germany) and stored at −20 °C until deoxyribonucleic acid (DNA) extraction.

### DNA extraction and real-time PCR

For further analysis, urine samples were submitted to IDEXX Laboratories (Ludwigsburg, Germany). Total nucleic acid was extracted from urine as previously described [25]. *Leptospira* real-time PCR was performed using LightCycler 480 (Roche, Mannheim, Germany) with proprietary forward, reverse primers, and hydrolysis probes. The target gene was *lipL32*/*hap1* (accession number AF245281.1), detecting only pathogenic *Leptospira*. This PCR had been shown to have a reproducible average analytical sensitivity of 10 DNA molecules per reaction.

### Urine culture and sequencing of culture-positive sample

Of each urine sample, 0.5 ml were cultured in Ellinghausen-McCullough-Johnson-Harris (EMJH) medium as described previously [71–73]. Within 2 h after collection, 0.5 ml of each urine sample were added to a tube with 5 ml of liquid EMJH medium plus 0.2 mg/ml 5-fluorouracil (5-FU) [74] to a final dilution of 1:10. After mixing and transferring 0.5 ml of the mixture to a second tube with EMJH medium plus 0.2 mg/ml 5-FU, a dilution of 1:100 was prepared. Cultures were stored at 24–28 °C and controlled for growth of *Leptospira* under dark field microscope for a total of 6 months [73] approximately every 7 days. In culture with growth of *Leptospira*, number of grown *Leptospira* was estimated by microscope, using a ×20 objective. A 1:10 dilution containing *Leptospira* was filtered with a 0.2 μm pore size filter (Corning® Sterile Syringe Filter; Corning Incorporated, Wiesbaden, Germany) and then transferred into 9 ml fresh EMJH medium. At a density of >200 *Leptospira*/field, a passage of the culture into 30 ml EMJH medium was performed. At late exponential phase of leptospiral growth, aliquots of purified *Leptospira* were frozen with 5% dimethylsulfoxid (DMSO) (Merck KGaA, Darmstadt, Germany) at −80 °C in Eppendorf tubes (Eppendorf AG, Hamburg, Germany) until DNA extraction. QIAamp DNA Mini Kit (Qiagen, Hilden, Germany) was used to extract leptospiral DNA, following the manufacturer’s instructions (QIAamp® DNA Mini and Blood Mini Handbook, Appendix B: Protocol for Cultured Cells; Qiagen, Hilden, Germany). The DNA extract was stored at −20 °C until further analysis. For phylogenetic analysis, *secY* sequencing was performed as described previously [75]. A Neighbor Joining Tree (Fig. 1) was constructed using the software MEGA 7.

### Microscopic agglutination test

MAT was performed as previously described [3]. Serum samples were tested for antibodies against 23 locally common pathogenic *Leptospira* serovars (Anhoa, Australis, Autumnalis, Ballum, Bataviae, Bratislava, Broomi, Canicola, Celledoni, Copenhageni, Coxi, Cynopteri, Djasiman, Grippotyphosa, Haemolytica, Icterohaemorrhagiae, Khorat, Paidjan, Pomona, Pyrogenes, Rachmati, Saxkoebing, Sejroe) and 1 saprophytic serovar Patoc, belonging to 15 serogroups (Australis, Autumnalis, Ballum, Bataviae, Canicola, Celledoni, Cynopteri, Djasiman, Grippotyphosa, Icterohaemorrhagiae, Javanica, Pomona, Pyrogenes, Sejroe, Semaranga) and 1 undesignated serogroup (Table 5). The cross-reacting strain Patoc I is of saprophytic origin and agglutination gives hints of an unidentified serovar not represented in the MAT panel. Two-fold dilutions of serum from 1:20 to 1:640 were tested. Threshold for reactivity was defined as ≥1:20. The titer was recorded as the last dilution in which ≥50% of the *Leptospira* agglutinated.

**Table 5:** List of *Leptospira* strains tested in microscopic agglutination test (MAT)

Genomospecies	Serogroup	Serovar	Strain
*Leptospira biflexa*	Semaranga	Patoc	Patoc I
*Leptospira borgpetersenii*	Ballum	Ballum	Mus 127
*Leptospira borgpetersenii*	Celledoni	Anhoa	LT 90–68
*Leptospira borgpetersenii*	Sejroe	Saxkoebing	Mus 24
*Leptospira borgpetersenii*	Sejroe	Sejroe	M 84
*Leptospira interrogans*	Australis	Australis	Ballico
*Leptospira interrogans*	Australis	Bratislava	Jez Bratislava
*Leptospira interrogans*	Autumnalis	Autumnalis	Akiyami A
*Leptospira interrogans*	Autumnalis	Rachmati	Rachmat
*Leptospira interrogans*	Bataviae	Bataviae	Swart
*Leptospira interrogans*	Bataviae	Paidjan	Paidjan
*Leptospira interrogans*	Canicola	Broomi	Patane
*Leptospira interrogans*	Canicola	Canicola	Hond Utrecht IV
*Leptospira interrogans*	Djasiman	Djasiman	Djasiman
*Leptospira interrogans*	Icterohaemorrhagiae	Copenhageni	M 20
*Leptospira interrogans*	Icterohaemorrhagiae	Icterohaemorrhagiae	RGA
*Leptospira interrogans*	Pomona	Pomona	Pomona
*Leptospira interrogans*	Pyrogenes	Pyrogenes	Salinem
*Leptospira interrogans*	Sejroe	Haemolytica	Marsh
*Leptospira kirschneri*	Cynopteri	Cynopteri	3522 C
*Leptospira kirschneri*	Grippotyphosa	Grippotyphosa	Moskva V
*Leptospira weilii*	Celledoni	Celledoni	Celledoni
*Leptospira weilii*	Javanica	Coxi	Cox
*Leptospira wolffii*	Undesignated	Khorat	Khorat H2

### IgM and IgG ELISA

For coating of the ELISA plates, a suspension of outer envelope antigen from 3 different strains (*Leptospira interrogans* serovar Canicola strain Hond Utrecht IV, serovar Icterohaemorrhagiae strain Kantorowicz, and serovar Copenhageni strain Wijnberg; produced by Leptospirosis Reference Centre, Amsterdam, the Netherlands) were used. The stock of antigen was diluted with PBS to a concentration of 2 μg/ml. Of this diluted antigen, 100 μl were added to every well. Incubation was performed overnight at room temperature. Coated plates were frozen at −20 °C until use. Dilution of all sera (controls and samples) from 1:20 to 1:2560 with dilution buffer (PBS + 1% protifar® (Nutricia Advanced Medical Nutrition, Zoetermeer, the Netherlands) + 0.05% Tween 80) was conducted twice; 1 dilution series for IgG and 1 for IgM antibodies was performed. The plates were covered with tape and incubated for 1 h at 37 °C in water bath. The incubated plates were rinsed 4 times with wash buffer (distilled water + 0.05% Tween 80), conjugate was added (Goat anti-Dog IgG and Goat anti-Dog IgM (Tebu-bio.com, Heerhugowaard, the Netherlands)) and mixed. Covered plates were incubated for 1 h at 37 °C in water bath. Afterwards, plates were rinsed 4 times with wash buffer (distilled water + 0.05% Tween 80), and 100 μl substrate (5.2 ml Na_2_HPO_4_ (Merck KGaA, Darmstadt, Germany), 4.8 ml citric acid (Merck KGaA, Darmstadt, Germany), 10 ml PBS, a 2,2′-azino-bis(3-ethylbenzothiazoline-6-sulfonic acid) diammonium salt tablet (10 mg; Merck KGaA, Darmstadt, Germany), and 10 μl H_2_O_2_ 30% (Merck KGaA, Darmstadt, Germany)) was added to every well. After 30 min, reading of plates was performed at room temperature. Positive and negative controls were included on each ELISA plate. The cut-off for reactivity was ≥1:320 in IgM and IgG ELISA.

### Risk factor analysis

Dog owners were requested to answer a standardized questionnaire in order to evaluate risk factors associated with *Leptospira* infection in dogs (Table 4). Lifestyle and activity parameters of the questionnaire were not recorded in case of stray dogs (*n* = 119). The approximate age of stray dogs was estimated based on dental examination. Health status of each dog was determined using a standardized physical examination protocol.

### Statistical analysis

For sample size calculation, an a priori power analysis using EpiTools epidemiological calculators (Ausvet, Australia) was performed to determine an appropriate sample size to achieve adequate power. Assuming an expected prevalence of urinary shedding of *Leptospira* (4.0%) with a 95% confidence interval (CI) of the estimation with a 5% precision, at least 236 dogs were necessary to achieve adequate power (>90.0%).

Statistical analysis to determine risk factors was performed with SPSS version 24 for Windows (IBM Cooperation, Armonk, USA). For univariate analysis, Fisher’s exact test was used to assess risk factors associated with *Leptospira* infection including all dogs with urinary shedding and/or presence of antibodies in MAT and/or in ELISA, (defined as “*Leptospira*-infected”) (Table 4). In addition, 2 risk factor analyses were performed for the following 2 subgroups: presence of antibodies against *Leptospira* determined by MAT (see Additional file 1: Table S1), and urinary shedding of *Leptospira* detected by PCR (see Additional file 2: Table S2). Multivariate logistic regression analysis was performed using parameters with at least 205 observations as independent variables, as available data of ≥75.0% of the dogs was chosen as mandatory criteria for entering. Backwards stepwise selection based on Wald was performed to detect the most important variables for being *Leptospira*-infected. Following parameters met the inclusion criteria: age; breed; sex; neutering status; origin; environment. A value of *p* <0.05 was considered significant.

## Supplementary information

**Additional file 1 : Table S1.** Risk factor analysis for dogs with MAT antibody titers (≥1:20) against *Leptospira.* Univariate and multivariate analysis for risk factors associated with MAT titers (cut-off: ≥1:20) in 33/273 dogs. For multivariate analysis, backward stepwise selection based on Wald was performed for the following categories: age, breed, sex, neutering status, origin, and environment.

**Additional file 2 : Table S2.** Risk factor analysis for dogs with urinary shedding of *Leptospira* determined by PCR. Univariate and multivariate analysis for risk factors associated with positive urine PCR results in 12/273 dogs. For multivariate analysis, backward stepwise selection based on Wald was performed for the following categories: age, breed, sex, neutering status, origin, and environment.

## Data Availability

The dataset supporting the results of the *secY* sequencing is deposited in GenBank at the National Center for Biotechnology Information (NCBI) https://www.ncbi.nlm.nih.gov/genbank/ under accession numbers MN862540-MN862558. Raw data (Excel file) is available from the corresponding author on request.

## Abbreviations

5-FU: 5-Fluorouracil
ATL: Animal tissue lysis
CI: Confidence interval
Ct: *Threshold cycle*
DMSO: Dimethylsulfoxid
DNA: Deoxyribonucleic acid
ELISA: Enzyme-linked immunosorbent assay
EMJH: Ellinghausen-McCullough-Johnson-Harris
IgG: Immunoglobulin G
IgM: Immunoglobulin M
OR: Odds ratio
*p*: *P*-value
PBS: Phosphate buffered saline
PCR: Polymerase chain reaction
