# Supplementary material for: Leptospira infection and shedding in dogs in Thailand
Source: BMC Vet Res. 2020 Mar 17;16:89. doi: 10.1186/s12917-020-2230-0 (PMC7077098; doi:10.1186/s12917-020-2230-0)
Supplement: Supplementary file 1 — Additional file 1 : Table S1. Risk factor analysis for dogs with MAT antibody titers (≥1:20) against Leptospira. Univariate and multivariate analysis for risk factors associated with MAT titers (cut-off: ≥1:20) in 33/273 dogs. For multivariate analysis, backward stepwise selection based on Wald was performed for the following categories: age, breed, sex, neutering status, origin, and environment. [file 12917_2020_2230_MOESM1_ESM.docx]

**Supplementary Table 1**

**Risk factor analysis for dogs with MAT antibody titers (≥1:20) against *Leptospira***

| **Variable** | **Total dogs** | **Categories** | **Number of dogs tested** | ***Leptospira-***  **positive** | ***Leptospira-* negative** | **Univariate**  **analysis** | | | **Multivariate**  **analysis**  **(n = 242)** | | |
| --- | --- | --- | --- | --- | --- | --- | --- | --- | --- | --- | --- |
|  |  |  |  | **(%)** | **(%)** | **Odds ratio** | **95% CI** | ***p*** | **Odds ratio** | **95% CI** | ***p*** |
| **Age** | **242** | <1 year | 36 | 3 (8.3) | 33 (91.7) | 0.339 | 0.087-1.316 | 0.142 | ^a^ | ^a^ | ^a^ |
|  |  | 1-1.9 years | 64 | 6 (9.4) | 58 (90.6) | 0.386 | 0.332-1.127 | 0.112 |  |  |  |
|  |  | 2-2.9 years | 45 | 4 (8.9) | 41 (91.1) | 0.364 | 0.110-1.236 | 0.166 |  |  |  |
|  |  | 3-3.9 years | 52 | 11 (21.2) | 41 (78.8) | Reference |  |  |  |  |  |
|  |  | 4-5.9 years | 28 | 6 (21.4) | 22 (78.6) | 1.017 | 0.331-3.120 | 1.000 |  |  |  |
|  |  | ≥6 years | 17 | 2 (11.8) | 15 (88.2) | 0.497 | 0.099-2.508 | 0.495 |  |  |  |
| **Breed** | **273** | mix | 266 | 32 (12.0) | 234 (88.0) |  |  |  | ^a^ | ^a^ | ^a^ |
|  |  | pure breed | 7 | 1 (14.3) | 6 (85.7) | 1.219 | 0.142-10.453 | 1.000 |  |  |  |
| **Sex** | **273** | female | 185 | 26 (14.1) | 159 (85.9) | 1.892 | 0.788-4.546 | 0.169 | ^a^ | ^a^ | ^a^ |
|  |  | male | 88 | 7 (8.0) | 81 (92.0) |  |  |  |  |  |  |
| **Neutering status** | **273** | intact | 270 | 32 (11.9) | 238 (88.1) |  |  |  | ^a^ | ^a^ | ^a^ |
|  |  | neutered | 3 | 1 (33.3) | 2 (66.7) | 3.719 | 0.328-42.184 | 0.322 |  |  |  |
| **Weight** | **175** | 5-11 kg | 48 | 6 (12.5) | 42 (87.5) | 1.057 | 0.359-3.115 | 1.000 |  |  |  |
|  |  | 12-17 kg | 84 | 10 (11.9) | 74 (88.1) | Reference |  |  |  |  |  |
|  |  | ≥18 kg | 43 | 6 (14.0) | 37 (86.0) | 1.200 | 0.405-3.556 | 0.781 |  |  |  |
| **Origin** | **273** | client-owned | 154 | 22 (14.3) | 132 (85.7) |  |  |  |  |  |  |
|  |  | stray | 119 | 11 (9.2) | 108 (90.8) | 0.611 | 0.284-1.316 | 0.262 | 0.514 | 0.236-1.119 | ^b^ 0.094 |
| **Environment** | **273** | urban | 134 | 18 (13.4) | 116 (86.6) | 1.283 | 0.618-2.663 | 0.579 | ^a^ | ^a^ | ^a^ |
|  |  | rural | 139 | 15 (10.8) | 124 (89.2) |  |  |  |  |  |  |
| **Free-running/ roaming allowed** | **180** | yes | 174 | 21 (12.1) | 153 (87.9) |  |  |  |  |  |  |
|  |  | no | 6 | 2 (33.3) | 4 (66.7) | 3.643 | 0.628-21.123 | 0.170 |  |  |  |
| **Variable** | **Total dogs** | **Categories** | **Number of dogs tested** | ***Leptospira-***  **positive** | ***Leptospira-* negative** | **Univariate**  **analysis** | | | **Multivariate**  **analysis**  **(n = 242)** | | |
|  |  |  |  | **(%)** | **(%)** | **Odds ratio** | **95% CI** | ***p*** | **Odds ratio** | **95% CI** | ***p*** |
| **Staying outdoors** | **168** | yes | 148 | 16 (10.8) | 132 (89.2) |  |  |  |  |  |  |
| **>50 %** |  | no | 20 | 3 (15.0) | 17 (85.0) | 1.456 | 0.384-5.519 | 0.704 |  |  |  |
| **Bathing in water** | **32** | yes | 13 | 2 (15.4) | 11 (84.6) |  |  |  |  |  |  |
|  |  | no | 19 | 5 (26.3) | 14 (73.7) | 1.964 | 0.318-12.124 | 0.671 |  |  |  |
| **Drinking out of puddles** | **34** | yes | 13 | 2 (15.4) | 11 (84.6) |  |  |  |  |  |  |
|  |  | no | 21 | 5 (23.8) | 16 (76.2) | 1.719 | 0.281-10.509 | 0.682 |  |  |  |
| **Contact with rodents** | **33** | yes | 22 | 5 (22.7) | 17 (77.3) | 1.324 | 0.213-8.235 | 0.764 |  |  |  |
|  |  | no | 11 | 2 (18.2) | 9 (81.8) |  |  |  |  |  |  |
| **Eating rodents** | **33** | yes | 6 | 3 (50.0) | 3 (50.0) | 5.750 | 0.843-39.241 | 0.093 |  |  |  |
|  |  | no | 27 | 4 (14.8) | 23 (85.2) |  |  |  |  |  |  |
| **Consumption of raw meat** | **40** | yes | 12 | 3 (25.0) | 9 (75.0) | 1.222 | 0.250-5.983 | 0.804 |  |  |  |
|  |  | no | 28 | 6 (21.4) | 22 (78.6) |  |  |  |  |  |  |
| **Hunting dog** | **273** | yes | 0 | 0 (0.0) | 0 (0.0) |  |  |  |  |  |  |
|  |  | no | 273 | 33 (12.1) | 240 (87.9) | 7.179 | 0.140-367.897 | 1.000 |  |  |  |
| **Contact with cats** | **50** | yes | 24 | 7 (29.2) | 17 (70.8) | 3.157 | 0.711-14.017 | 0.164 |  |  |  |
|  |  | no | 26 | 3 (11.5) | 23 (88.5) |  |  |  |  |  |  |
| **Contact with other dogs** | **176** | yes | 175 | 22 (12.6) | 153 (87.4) | 2.274 | 0.090-57.551 | 1.000 |  |  |  |
|  |  | no | 1 | 0 (0.0) | 1 (100.0) |  |  |  |  |  |  |
| **Contact with cattle** | **58** | yes | 16 | 2 (12.5) | 14 (87.5) |  |  |  |  |  |  |
|  |  | no | 42 | 8 (19.0) | 34 (81.0) | 1.647 | 0.310-8.748 | 0.710 |  |  |  |
| **Contact with pigs** | **58** | yes | 1 | 1 (100.0) | 0 (0.0) | 15.316 | 0.579-405.137 | 0.173 |  |  |  |
|  |  | no | 57 | 9 (15.8) | 48 (84.2) |  |  |  |  |  |  |

Univariate and multivariate analysis for risk factors associated with MAT titers (cut-off: ≥1:20) in 33/273 dogs. For multivariate analysis, backward stepwise selection based on Wald was performed for the following categories: age, breed, sex, neutering status, origin, and environment.

^a^Variable was eliminated in backward stepwise selection

^b^Not significant, *p*-value only showed a tendency

*MAT* microscopic agglutination test, *CI* confidence interval, *p* = *p*-value
