# Supplementary material for: Leptospira infection and shedding in dogs in Thailand
Source: BMC Vet Res. 2020 Mar 17;16:89. doi: 10.1186/s12917-020-2230-0 (PMC7077098; doi:10.1186/s12917-020-2230-0)
Supplement: Supplementary file 2 — Additional file 2 : Table S2. Risk factor analysis for dogs with urinary shedding of Leptospira determined by PCR. Univariate and multivariate analysis for risk factors associated with positive urine PCR results in 12/273 dogs. For multivariate analysis, backward stepwise selection based on Wald was performed for the following categories: age, breed, sex, neutering status, origin, and environment. [file 12917_2020_2230_MOESM2_ESM.docx]

**Supplementary Table 2**

**Risk factor analysis for dogs with urinary shedding of *Leptospira* determined by PCR**

| **Variable** | **Total dogs** | **Categories** | **Number of dogs tested** | ***Leptospira-***  **positive** | ***Leptospira-* negative** | **Univariate**  **analysis** | | | **Multivariate**  **analysis**  **(n = 242)** | | |
| --- | --- | --- | --- | --- | --- | --- | --- | --- | --- | --- | --- |
|  |  |  |  | **(%)** | **(%)** | **Odds ratio** | **95% CI** | ***p*** | **Odds ratio** | **95% CI** | ***p*** |
| **Age** | **242** | <1 year | 36 | 1 (2.8) | 35 (97.2) | 0.343 | 0.037-3.202 | 0.403 | ^a^ | ^a^ | ^a^ |
|  |  | 1-1.9 years | 64 | 2 (3.1) | 62 (96.9) | 0.387 | 0.068-2.203 | 0.406 |  |  |  |
|  |  | 2-2.9 years | 45 | 3 (6.7) | 42 (93.3) | 0.857 | 0.181-4.051 | 1.000 |  |  |  |
|  |  | 3-3.9 years | 52 | 4 (7.7) | 48 (92.3) | Reference |  |  |  |  |  |
|  |  | 4-5.9 years | 28 | 2 (7.1) | 26 (92.9) | 0.923 | 0.158-5.383 | 1.000 |  |  |  |
|  |  | ≥6 years | 17 | 0 (0.0) | 17 (100.0) | 0.308 | 0.016-6.017 | 0.565 |  |  |  |
| **Breed** | **273** | mix | 266 | 11 (4.1) | 255 (95.9) |  |  |  | ^a^ | ^a^ | ^a^ |
|  |  | pure breed | 7 | 1 (14.3) | 6 (85.7) |  |  |  |  |  |  |
| **Sex** | **273** | female | 185 | 9 (4.9) | 176 (95.1) | 1.449 | 0.382-5.4651 | 0.757 | ^a^ | ^a^ | ^a^ |
|  |  | male | 88 | 3 (3.4) | 85 (96.6) |  |  |  |  |  |  |
| **Neutering status** | **273** | intact | 270 | 12 (4.4) | 258 (95.6) | 2.954 | 0.145-60.363 | 0.482 | ^a^ | ^a^ | ^a^ |
|  |  | neutered | 3 | 0 (0.0) | 3 (100.0) |  |  |  |  |  |  |
| **Weight** | **175** | 5-11 kg | 48 | 4 (8.3) | 44 (91.7) | 2.445 | 0.526-11.465 | 0.421 |  |  |  |
|  |  | 12-17 kg | 84 | 3 (3.6) | 81 (96.4) | Reference |  |  |  |  |  |
|  |  | ≥18 kg | 43 | 3 (7.0) | 40 (93.0) | 2.025 | 0.391-10.487 | 0.663 |  |  |  |
| **Origin** | **273** | client-owned | 154 | 5 (3.2) | 149 (96.8) |  |  |  | ^a^ | ^a^ | ^a^ |
|  |  | stray | 119 | 7 (5.9) | 112 (94.1) | 1.863 | 0.576-6.022 | 0.375 |  |  |  |
| **Environment** | **273** | urban | 134 | 4 (3.0) | 130 (97.0) |  |  |  | ^a^ | ^a^ | ^a^ |
|  |  | rural | 139 | 8 (5.8) | 131 (94.2) | 1.985 | 0.583-6.753 | 0.378 |  |  |  |
| **Free-running/ roaming allowed** | **180** | yes | 174 | 9 (5.2) | 165 (94.8) | 1.340 | 0.07-25.601 | 1.000 |  |  |  |
|  |  | no | 6 | 0 (0.0) | 6 (100.0) |  |  |  |  |  |  |
| **Variable** | **Total dogs** | **Categories** | **Number of dogs tested** | ***Leptospira-***  **positive** | ***Leptospira-* negative** | **Univariate**  **analysis** | | | **Multivariate**  **analysis**  **(n = 242)** | | |
|  |  |  |  | **(%)** | **(%)** | **Odds ratio** | **95% CI** | ***p*** | **Odds ratio** | **95% CI** | ***p*** |
| **Staying outdoors** | **168** | yes | 148 | 7 (4.7) | 141 (95.3) |  |  |  |  |  |  |
| **>50 %** |  | no | 20 | 2 (10.0) | 18 (90.0) | 2.238 | 0.431-11.610 | 0.291 |  |  |  |
| **Bathing in water** | **32** | yes | 13 | 2 (15.4) | 11 (84.6) | 8.478 | 0.373-192.510 | 0.157 |  |  |  |
|  |  | no | 19 | 0 (0.0) | 19 (100.0) |  |  |  |  |  |  |
| **Drinking out of puddles** | **34** | yes | 13 | 2 (15.4) | 11 (84.6) | 9.348 | 0.413-211.632 | 0.139 |  |  |  |
|  |  | no | 21 | 0 (0.0) | 21 (100.0) |  |  |  |  |  |  |
| **Contact with rodents** | **33** | yes | 22 | 2 (9.1) | 20 (90.9) | 2.805 | 0.124-63.591 | 0.542 |  |  |  |
|  |  | no | 11 | 0 (0.0) | 11 (100.0) |  |  |  |  |  |  |
| **Eating rodents** | **33** | yes | 6 | 1 (16.7) | 5 (83.3) | 5.200 | 0.277-97.617 | 0.335 |  |  |  |
|  |  | no | 27 | 1 (3.7) | 26 (96.3) |  |  |  |  |  |  |
| **Consumption of raw meat** | **40** | yes | 12 | 1 (8.3) | 11 (91.7) | 2.455 | 0.141-42.824 | 0.515 |  |  |  |
|  |  | no | 28 | 1 (3.6) | 27 (96.4) |  |  |  |  |  |  |
| **Hunting dog** | **273** | yes | 0 | 0 (0.0) | 0 (0.0) |  |  |  |  |  |  |
|  |  | no | 273 | 12 (4.4) | 261 (95.6) | 20.920 | 0.399-1098.352 | 1.000 |  |  |  |
| **Contact with cats** | **50** | yes | 24 | 1 (4.2) | 23 (95.8) | 1.087 | 0.064-18.402 | 1.000 |  |  |  |
|  |  | no | 26 | 1 (3.8) | 25 (96.2) |  |  |  |  |  |  |
| **Contact with other dogs** | **176** | yes | 175 | 9 (5.1) | 166 (94.9) |  |  |  |  |  |  |
|  |  | no | 1 | 0 (0.0) | 1 (100.0) | 5.842 | 0.223-153.216 | 1.000 |  |  |  |
| **Contact with cattle** | **58** | yes | 16 | 1 (6.2) | 15 (93.8) | 2.733 | 0.161-46.515 | 0.479 |  |  |  |
|  |  | no | 42 | 1 (2.4) | 41 (97.6) |  |  |  |  |  |  |
| **Contact with pigs** | **58** | yes | 1 | 0 (0.0) | 1 (100.0) |  |  |  |  |  |  |
|  |  | no | 57 | 2 (3.5) | 55 (96.5) | 7.400 | 0.237-231.338 | 1.000 |  |  |  |

Univariate and multivariate analysis for risk factors associated with positive urine PCR results in 12/273 dogs. For multivariate analysis, backward stepwise selection based on Wald was performed for the following categories: age, breed, sex, neutering status, origin, and environment.

^a^Variable was eliminated in backward stepwise selection

*PCR* polymerase chain reaction, *CI* confidence interval, *p* *p*-value
